# Methyl-CpG-binding domain protein 2 contributes to renal fibrosis through promoting polarized M1 macrophages

**DOI:** 10.1038/s41419-022-04577-3

**Published:** 2022-02-08

**Authors:** Kai Ai, Jian Pan, Pan Zhang, Huiling Li, Zhibiao He, Hongliang Zhang, Xiaozhou Li, Yijian Li, Lei Yi, Ye Kang, Yinhuai Wang, Xudong Xiang, Xiangping Chai, Dongshan Zhang

**Affiliations:** 1 Department of Emergency Medicine, Second Xiangya Hospital, Central South University, Changsha, China; 2 Emergency Medicine and Difficult Diseases Institute, Second Xiangya Hospital, Central South University, Changsha, China; 3 Department of Urology, Second Xiangya Hospital, Central South University, Changsha, China; 4 Hunan Provincial Key Laboratory of Clinical Epidemiology, Xiangya School of Public Health, Central South University, Changsha, China; 5 Department of Ophthalmology, Second Xiangya Hospital, Central South University, Changsha, China

**Keywords:** DNA methylation, DNA-binding proteins

## Abstract

Recent studies reported that Methyl-CpG–binding domain protein 2 (MBD2) promoted M2 macrophages accumulation to increase bleomycin-induced pulmonary fibrosis. However, the role and mechanism of action of MBD2 in macrophages differentiation and renal fibrosis remain largely unknown. In the current study, MBD2 not only promoted the differentiation of resting M0 macrophages to polarized M2 macrophages, but also induced them to polarized M1 macrophages and the transition of M2 to M1 macrophages. ChIP analysis demonstrated that MBD2 physically interacted with the promoter region of the CpG islands of G0S2 genes, and then activated their expression by inducing hypomethylation of the promoter region. Interestingly, the data demonstrated that the role of G0S2 in macrophages differentiation is consistent with MBD2. Furthermore, Co-culture of activated M1 macrophages and murine embryonic NIH 3T3 fibroblasts indicated that MBD2 mediated the M1-induction of ECM production by embryonic NIH 3T3 fibroblasts via promotion of G0S2. In addition, we also found that inhibition of MBD2 suppressed LPS induced the expression of p53 as well as activation and expression of stat3 in RAW264.7 macrophages. In vivo, MBD2 LysM^cre^ attenuated unilateral ureteral obstruction (UUO) and ischemia/reperfusion (I/R)-induced renal fibrosis via downregulation of G0S2, which was demonstrated by the downregulation of fibronectin (FN), collagen I and IV, α-SMA, G0S2. These data collectively demonstrated that MBD2 in macrophages contributed to UUO and I/R-induced renal fibrosis through the upregulation of G0S2, which could be a target for treatment for chronic kidney disease.

## Introduction

Renal fibrosis is a final common feature of chronic kidney disease (CKD) that eventually results in the loss of renal function [1]. The growing evidence demonstrated that Macrophages are involved in the progression of renal fibrosis [2–6]. Interestingly, activated M1 produced pro-inflammatory factors to induce renal injury, by contrast, activated M2 suppressed the inflammation to repair the kidney [7, 8]. It was a key scientific question to investigate the mechanism of activation and polarization of macrophages for the treatment of renal fibrosis.

DNA methylation is thought to be “read” by a family of methyl-CpG-binding domain (MBD) proteins that includes MeCP2 and MBD1–4 [9]. Two studies suggested that MeCP2 regulated the gene expression of macrophages [10, 11]. A more recent study reported that MBD2 promoted the differentiation of resting M0 macrophages to polarized M2 macrophages and then induced bleomycin-induced pulmonary fibrosis [12]. As we know, M2 macrophages promote the progression of lung fibrosis [13, 14], but it has protective effect on renal fibrosis [7, 8]. Hence, we have to reevaluate the role and mechanism of MBD2 in the differentiation of resting M0 macrophages and renal fibrosis.

Recently, one study has reported that the G0/G1 switch protein 2 (G0S2) mediated the production of renal inflammation in chronic kidney disease (CKD) [15]. Interestingly another study reported that 5-Aza-2′-deoxycytidine, an inhibitor of DNA methylation, restored the G0S2 expression in squamous cell lung cancer cell lines [16], which suggested that DNA methylation might regulate G0S2 expression. Thus, the role and mechanism of regulation for MBD2 in the expression of G0S2 remain unclear. We hypothesized that MBD2 in macrophages might promote the differentiation of resting M0 macrophages to mediate the renal fibrosis by upregulating the expression of G0S2.

In this study, we verified that inhibition of MBD2 in macrophages attenuated TGF-β1- and UUO and I/R-induced renal fibrosis. We also discovered the molecular mechanism underlying MBD2-induced renal fibrosis in UUO and ischemic injury via upregulation of the expression of G0S2.

## Materials and methods

### Reagents and antibodies

Antibodies were obtained from multiple sources: anti-GAPDH from Santa Cruz Biotechnology (Santa Cruz, CA, USA), anti-MBD2, anti-collagen I, anti-α-SMA and anti-fibronectin from Abcam (Cambridge, MA, USA), and anti-F4/80, anti-collagen IV, and anti-G0S2 from Proteintech Group (Rosemont, IL, USA). The recombinant human TGF-β1 was obtained from Proteintech Group. The plasmids containing the methylation promoter of the G0S2 CpG-free pCpGI luciferase reporter, MBD2, and mtMBD2 (the deletion of the methylated DNA binding domain), were constructed by the Ruqi Biology Company (Guangdong, Guangzhou, China) according to previously published reports [15, 17]. The MBD2 siRNA was designed and synthesized by the Ruibo Biology Company (Guangdong, Guangzhou, China) as described in our previously published paper [18].

### Animals

The MBD2-LysM^Cre^ mice were generated by crossing MBD2^flox/flox^LysM^WT/WT^ (obtained from Shanghai Model Organisms) males with MBD2^+/+^LysM^WT/Cre^ females (obtained from Shanghai Model Organisms). C57BL/6 male mice aged 8–10 weeks were purchased from Hunan SJA Laboratory Animal Co., Ltd. We use the PASS Software to estimate sample size. The experimental mice were grouped by a simple random sampling method and observation was performed blindly. The unilateral ureteral obstruction (UUO) model was produced by ligating the left ureter, as previously described [19–21]. The ischemic acute kidney injury (I/R) model was induced by the duration of bilateral clamping for 28 min and followed by reperfusion for 21 days [22]. Animal experiments were carried out according to the guidelines of the Institutional Committee for the Care and Use of Laboratory Animals of the Second Xiangya Hospital, the People’s Republic of China, and authorized by the Institutional Animal Use Committee of Second Xiangya Hospital (IRB number is 20200320). The mice were housed under a 12-h light/dark cycle and given free access to food and drinking water.

### Cell culture and treatments

RAW264.7 macrophage was purchased from Shanghai Zhong Qiao Xin Zhou Biotechnology Co., Ltd. Cells were cultured with 10% fetal bovine serum (FBS)and 0.5% penicillin in Dulbecco’s modified Eagle’s medium (Thermo Fisher Scientific), and streptomycin in a 5% CO_2_ incubator at 37 °C. Twenty-four hours after transfection with MBD2 siRNA, a negative control, or MBD2 plasmid, were subjected to nutrient deprivation in a serum-free medium overnight. Then the cells were or were not treated with LPS (1 μg/ml) or IL-4 (20 ng/ml) for an additional 24 h.

### Cell isolation and differentiation

Primary bone marrow-derived macrophages were isolated, as described previously [23]. Briefly, bone marrow cells were obtained by flushing the mouse femur using RPMI 1640 supplemented with 10% heat-inactivated FBS and then treated with RBC lysis buffer. Cell suspensions (3 × 10^6^ cells per well) were cultured in dishes using complete medium supplemented with recombinant murine M-CSF (20 ng/ml, Peprotech). Subsequently, the cell concentration was adjusted to culture for six days to obtain M0 macrophages. The M0 macrophages were stimulated with 1 μg/ml LPS or IL-4 20 ng/ml IL-4 to cause polarization into M1 or M2 macrophages, respectively.

### Kidney-infiltrating mononuclear cells (KMNCs) isolation

C57BL/6 or MBD2-LysMCre mice were first exsanguinated and euthanized by the dislocation of cervical vertebra. Subsequently, kidneys were taken out from mice and then digested in RPMI 1640 medium containing 2 mg/ml collagenase IV (GIBCO) and 100 mg/ml DNase I (Roche) for 45 min at 37 °C. After tissue disaggregation, cells were filtered through a 70 µm cell strainer and washed with PBS. As described previously [24, 25], 36 and 72% Percoll solution (Amersham Pharmacia) was used to separate renal immune cells, which were counted, and then used for flow cytometry.

### Flow cytometry

Flow cytometry was performed using standard protocols, as described previously [26]. The kidney and spleen of mice were obtained, and single-cell suspensions were prepared. The single cells were treated with Fc block (BD Biosciences, SanJose, CA, USA) and incubated with the following fluorescent antibodies [27]; CD11b-FITC, CD45–Percp, F480-PE, and CD11c-PE-CY7 for 30 min at 4 °C, then washed and fixed according to the manufacturer’s protocol (BD Cytofix; BD Biosciences). Some of the cell suspensions were stained with Fc block and an intracellular antibody, CD206-APC, for intracellular flow cytometry [27]. Flow cytometry was performed on a FACS Canto II (BD Biosciences), and the data were analyzed using FlowJo, version 10.2.

### ChIP analysis and transcriptional activation activity

Chromatin immunoprecipitation (ChIP) assays were performed using anti-MBD2 according to the protocol described for the ChIP kit (Millipore, USA) [18, 21, 28–30]. Precipitated DNA was amplified by PCR using CpG island binding primers for promoters of EGR1 and G0S2. G0S2: F1: 5′-TTGTTTGATAAGGTTAAAAGAA-3′, R1: 5′-ACAATCTTACAACAACCTTTACAAT-3′; F2: 5′-TTGTTTGATAAGGTTAAAAGAA-3′, R2: 5′-ATACAATCTTACAACAACCTTTACAAT-3′; F3: 5′-TTGTTTGATAAGGTTAAAAGAA-3′, R3: 5′-TACAATCTTACAACAACCTTTACAAT-3′; F4: 5′-TTGTTTGATAAGGTTAAAAGAA-3′, R5: 5′-AAAATTACAAAAATCAATAATACAATCTTA-3′; F5: 5′-TTGTTTGATAAGGTTAAAAGAA-3′, R5: 5′-CAATCTTACAACAACCTTTACAAT-3′. The transcriptional activation activity of and G0S2 was evaluated using the relative activity of luciferase by employing the Promega kit as described previously [31].

### Methylated CpG-DNA immunoprecipitation

The methylated CpG-DNA immunoprecipitation assay was carried out according to the manufacturer’s instructions (Zymo Research) as previously described [32]. Briefly, purified genomic DNA was used for methylated CpG immunoprecipitation and subjected to real-time PCR analysis.

### Histology, Immunohistochemistry, immunofluorescence and Immunoblot Analyses

Harvested kidney tissues were fixed in 4% buffered paraformaldehyde, embedded in paraffin blocks, and sectioned at 4 μm thickness. Sections were stained with hematoxylin and eosin (HE) and Masson’s trichrome stains [21]. Immunohistochemical analyses were carried out using anti-MBD2, collagen I, collagen IV and fibronectin according to previously published protocols [21]. The immunofluorescence of MBD2 and F4/80 was carried out following the standard procedure [22]. The details for quantification have been described in our recently published study [19]. For Western blot analysis, tissue lysates from kidneys or cells were subjected to SDS-PAGE gel electrophoresis, membrane transfer, and immunodetection using anti-MBD2, collagen I, collagen IV, fibronectin, and G0S2 antibodies according to published standard procedures.

### Real-time polymerase chain reaction

Real-time quantitative reverse transcription PCR amplification was carried out according to previously described protocols [30]. The following primer pairs were used: TNF-α: forward 5′-CAGGCGGT GCCTATGTCTC-3′, reverse 5′-CGATCACCCCGAAGTTCAGTAG-3′; IL-1β: forward 5′-GAAATGCCACCTTTTGACAGTG-3′, reverse 5′- CTGGATGCTCTCATCAGGACA-3′; TGF-β1: forward 5′-GAGCCCGAAGCGGACTACTA-3′, reverse 5′-GTTGTTGCGGTCCAC CATT-3′; Arg1: forward 5′-CTCCAAGCCAAAGTCCTTAGAG-3′, reverse 5′-GGAGCT GTCATTAGGGACATCA-3′; GADPH: forward 5′-AGGTCGGTGTGAACGGATTTG-3′, reverse 5′-GGGGTCGTTGATGGCAACA-3′.

### Statistical analysis

Data were expressed as means ± SD. Student’s *t*-test was used for comparison between two groups. One-way ANOVA followed by Tukey’s post hoc analysis was used for multiple comparisons. The Kruskal–Wallis test was used the data with non-normal distribution. Statistical significance was set at *P* < *0.05*.

## Result

### UUO induced the expression of MBD2 and numbers of M1 and M2 macrophages in kidney

**Fig. 1:**
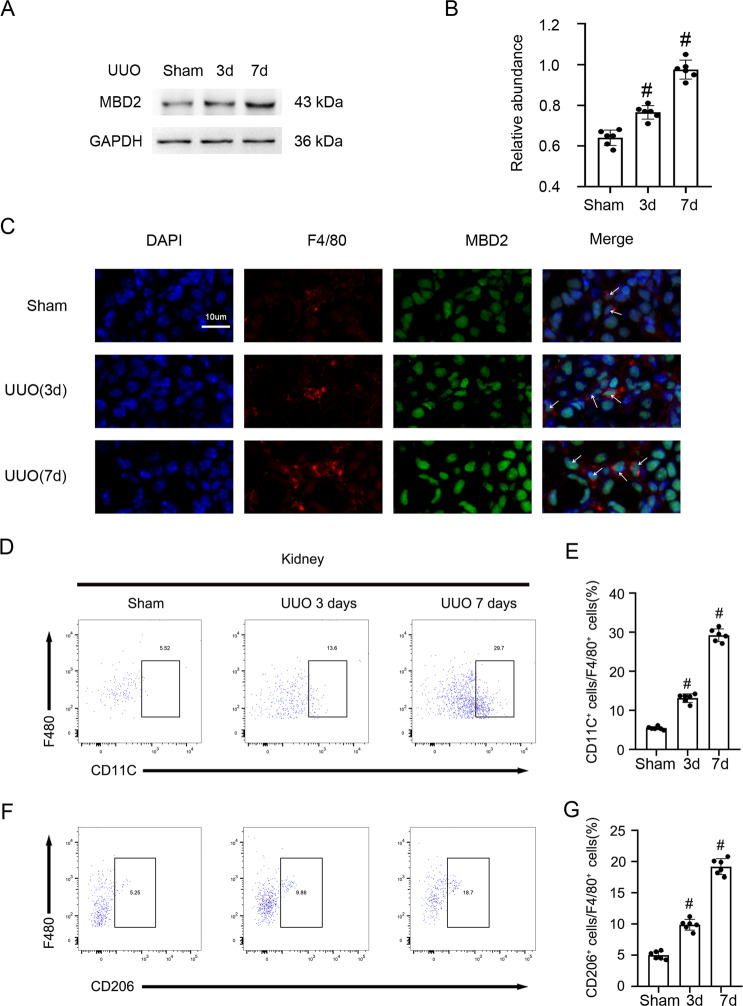
UUO induced the expression of MBD2 and M1 and M2 macrophages in kidney. Male C57BL/6 mice were subjected to UUO for examination on days 0–7. **A** The immunoblot of MBD2 and GAPDH. **B** Densitometry analysis of proteins levels, and normalized to internal control of GAPDH. **C** The immunofluorescence double staining of F4/80 and MBD2 in mice kidney. **D** and **E** Representative FCM analysis the ration of M1 (CD11C^+^ cells vs. F4/80 cells). **F** and **G** Representative FCM analysis the ration of M2 (CD206^+^ cells vs. F4/80 cells). Data are expressed as means ± sd (*n* = 6). ^#^*P* < 0.05 versus Shan group. Original magnification, x400.

We explored whether MBD2 was induced after treatment with UUO in C57BL/6 mice. We demonstrated that MBD2 in kidney cortex gradually was increased at days 3 after UUO, and then reached a peak at days 7 after UUO (Fig. 1A, B). Further, the immunofluorescence double staining of MBD2 and F4/80 indicated that staining signal of MBD2 and F4/80 was upregulated in the mouse UUO model at days three and seven (Fig. 1C). At the same time, the FCM analysis indicated the number of M1 and M2 macrophages in the kidney increased which supports the expression of F4/80 (Fig. 1D–G). Collectively, these data suggested that UUO induced the expression of MBD2 and numbers of M1 and M2 macrophages in kidney.

### MBD2 promoted the differentiation of M0 macrophages to M1 or M2 macrophages, and M2 to M1 macrophages

**Fig. 2:**
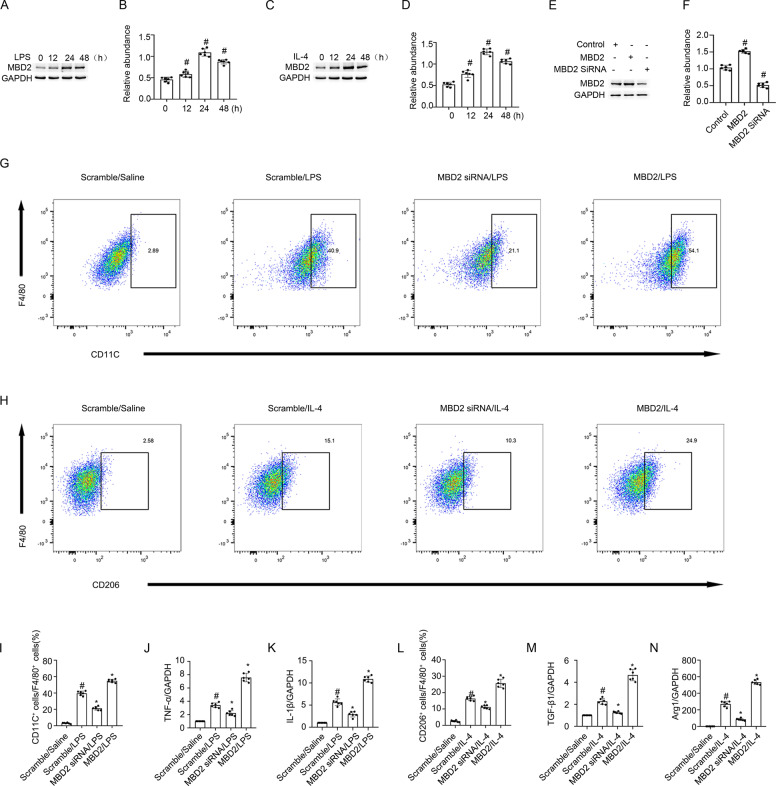
MBD2 promoted transition of M0 macrophages to M1 or M2. RAW264.7 macrophage cells were transfected with MBD2 siRNA or MBD2 plasmid plus with LPS 1 μg/ml or IL-4 20 ng/ml treatment for 24 h. **A**, **C,** and **E** The immunoblot of MBD2 and GAPDH. **B**, **D**, and **F** Densitometry analysis of proteins levels, and normalized to internal control of GAPDH. **G** and **I** Representative FCM analysis the ration of M1 (CD11C^+^ cells vs. F4/80 cells). **H** and **L** Representative FCM analysis the ration of M2 (CD206^+^ cells vs. F4/80 cells). **J** The expression levels of TNF-α were detected by real-time qPCR. **K** The expression levels of IL-1β were detected by real-time qPCR. **M** The expression levels of TGF-β1 were detected by real-time qPCR. **N** The expression levels of Arg1 were detected by real-time qPCR. Data are expressed as means ± sd (*n* = 6). ^#^*P* < *0.05* versus Scramble. *P < *0.05* versus LPS or IL-4 group.

**Fig. 3:**
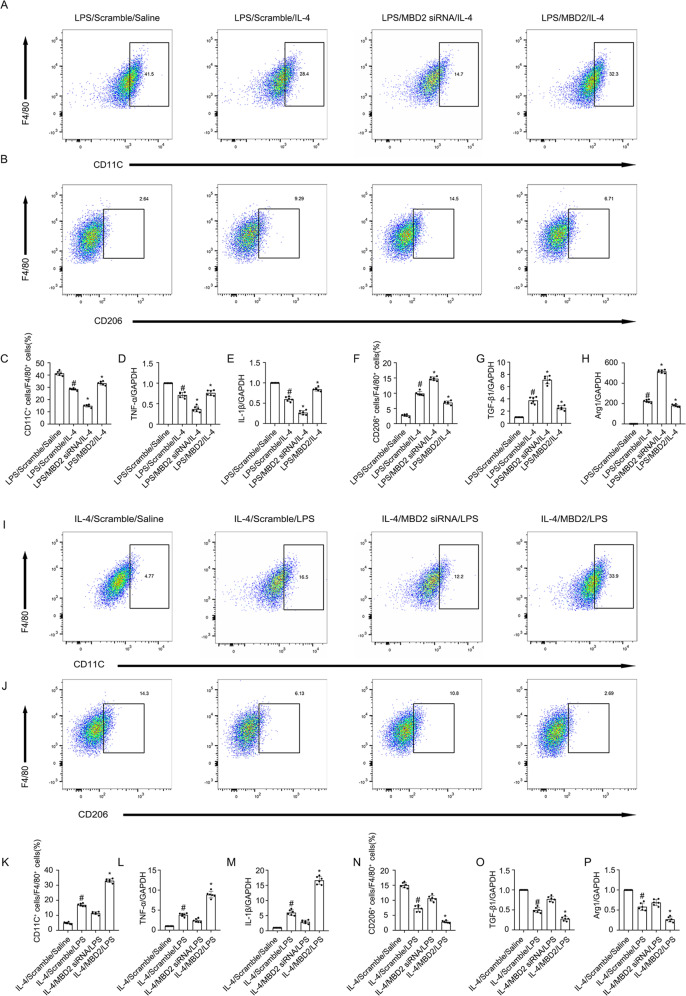
MBD2 promoted transition of M2 to M1. RAW264.7 macrophage cells were transfected with MBD2 siRNA or MBD2 plasmid plus with LPS 1 μg/ml or IL-4 20 ng/ml treatment for 24 h. **A**, **C**, **I**, and **K** Representative FCM analysis the ration of M1 (CD11C^+^ cells vs. F4/80 cells). **B**, **F**, **J**, and **N** Representative FCM analysis the ration of M2 (CD206^+^ cells vs. F4/80 cells). **D** and **L** The expression levels of TNF-α were detected by real-time qPCR. **E** and **M** The expression levels of IL-1β were detected by real-time qPCR. **G** and **O** The expression levels of TGF-β1 were detected by real-time qPCR. **H** and **P** The expression levels of Arg1 were detected by real-time qPCR. ^#^*P* < *0.05* versus Scramble plus LPS or IL-4 group. Data are expressed as means ± sd (*n* = 6). *P < *0.05* versus LPS and IL-4 group.

Macrophages play a critical role in UUO injury [33]. In this study, RAW264.7 macrophages were treated with LPS or IL-4. The Western blot results indicated that both LPS and IL-4 induced MBD2 expression that was initiated at 12 h, peaked at 24 h, and then decreased by 48 h (Fig. 2A–D). However, the role of MBD2 in macrophage differentiation remained unclear. To verify whether the MBD2 plasmid or siRNA were effective, they were transfected into RAW264.7 macrophages. The results indicated that MBD2 expression was enhanced by the MBD2 plasmid and reduced by MBD2 siRNA (Fig. 2E, F). The RAW264.7 macrophages were transfected with MBD2 siRNA or MBD2 plasmid with either LPS or IL-4. The flow cytometry (FCM) analysis indicated that LPS or IL-4 induced differentiation of M0 macrophages into M1 or M2 macrophages, respectively, which was blocked by MBD2 siRNA. In contrast, this effect was enhanced by overexpression of MBD2 (Fig. 2G, H, I, and L). The RT-qPCR analysis of TNF-α and IL-1β (M1 markers), as well as TGF-β1 and Arg1 (M2 markers) further supported the FCM results (Fig. 2J, K, M, and N). We investigated the role of MBD2 in the differentiation of macrophages from M1 to M2 and M2 to M1. After LPS or IL-4 treatment, the RAW264.7 macrophages were differentiated into M1 or M2 macrophages and then transfected with MBD2 siRNA or MBD2 plasmid and exposed to IL-4 or LPS. The FCM analysis and RT-qPCR results demonstrated that the differentiation of macrophages from M1 to M2 was enhanced by the MBD2 siRNA. In contrast, the effect was blocked by the overexpression of MBD2 (Fig. 3A–H). The FCM analysis and RT-qPCR results demonstrated that the differentiation of macrophages from M2 to M1 was prevented by the MBD2 siRNA. On the other hand, the effect was reinforced by the overexpression of MBD2 (Fig. 3I–P). Therefore, these data suggested that MBD2 induced the differentiation of M0 macrophages into M1 and M2 macrophages and the transition of M2 macrophages into M1 macrophages.

### MBD2 activated the expression of G0S2 by promoter demethylation

**Fig. 4:**
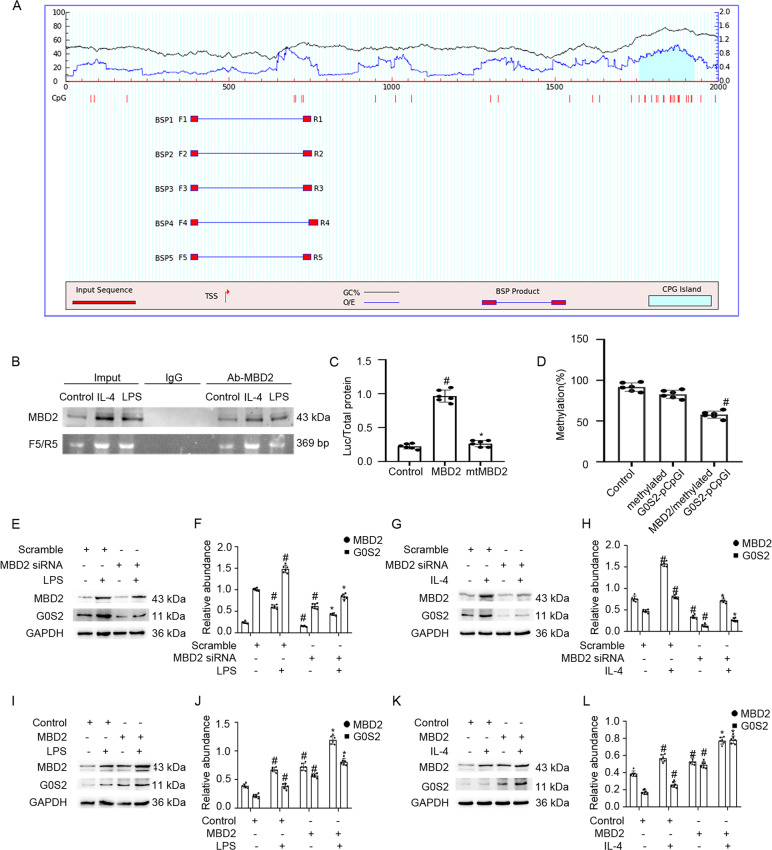
MBD2 directly binds to CpG islands of promoter of G0S2 and positively activates transcription of them by hypomethylation of promoter. **A** The prediction patterns of CpG islands of G0S2 promoter and five pairs of primer were shown using the software of MethPrimer 2.0. **B** ChIP assays represent the binding sites of MBD2 interaction with CpG islands of the promoter of G0S2 under LPS or IL-4 treatment conditions. **C** Relative luciferase activity of MBD2 or MBD2 mutation plasmids co-transfected with methylated G0S2 pCpGI plasmid in M0 macrophages. **D** CpG-DNA methylation level of the G0S2 promoter region. **E**, **G**, **I**, and **K** Representative immunoblots of MBD2 and G0S2. **F**, **H**, **J**, and **L** Densitometry analysis of proteins levels, and *normalized* to internal control of GAPDH. Data are expressed as means ± sd (*n* = 6). ^#^*P* < *0.05* versus Scramble or Control group. *P < *0.05* versus LPS or IL-4 group.

We assessed how MBD2 regulated the expression of G0S2. The results indicated that the promoter sequence of G0S2 existed at one island, according to the MethPrimer Promoter 2.0 (http://www.urogene.org/cgi-bin/methprimer2/MethPrimer.cgi) (Fig. 4A). As shown in Fig. 4B, ChIP analysis demonstrated that MBD2 physically interacted with the binding sites of F/R5-5 of the G0S2 promoter with LPS or IL-4 exposure. Furthermore, MBD2 but not the mutant plasmid of the MBD2 methylated DNA binding domain deletion enhanced the transcriptional activity of the CpG-free pCpGI luciferase reporter. The plasmid contained the promoter methylation region of G0S2 (Fig. 4C). The methylated pCpGI of G0S2 was somewhat suppressed by endogenous MBD2-bound DNA and was markedly inhibited by ectopic MBD2 expression (Fig. 4D). While G0S2 was induced by LPS or IL-4, it was reduced by exposure to the MBD2 siRNA. In contrast, this effect was enhanced by the MBD2 plasmid (Fig. 4E–L). These data demonstrated that MBD2 activated the expression of G0S2 through hypomethylation of the promoter region.

### G0S2 promoted the transition of M0 macrophages to M1 or M2 macrophages, and M2 to M1 macrophages

**Fig. 5:**
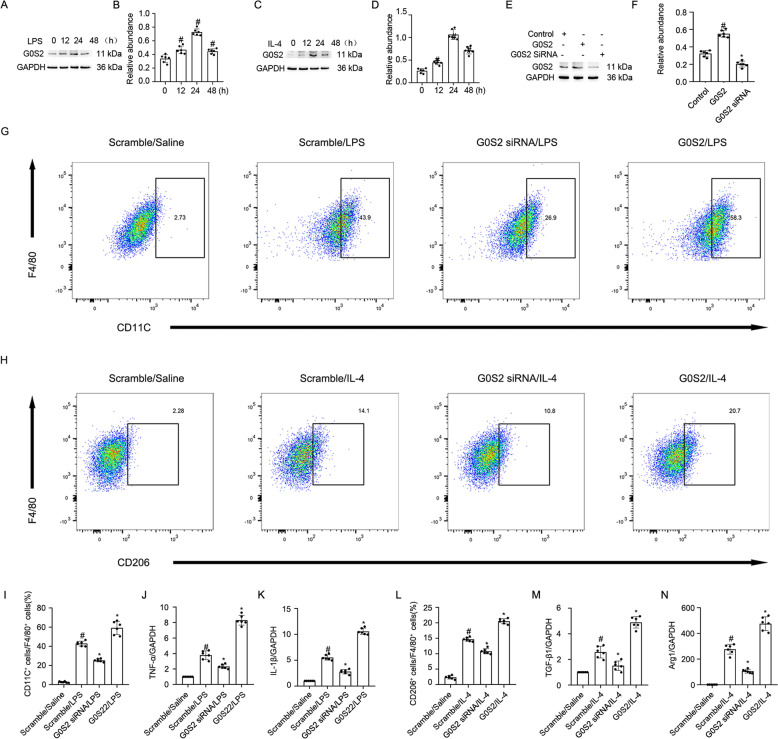
G0S2 promoted transition of M0 macrophages to M1 or M2. RAW264.7 macrophage cells were transfected with G0S2 siRNA or G0S2 plasmid plus with LPS 1 μg/ml or IL-4 20 ng/ml treatment for 24 h. **A**, **C**, and **E** Representative immunoblots of G0S2 and GAPDH. **B**, **D**, and **F** Densitometry analysis of proteins levels, and normalized to internal control of GAPDH. **G** and **I** Representative FCM analysis the ration of M1 (CD11C^+^ cells vs. F4/80 cells). **H** and **L** Representative FCM analysis the ration of M2 (CD206^+^ cells vs. F4/80 cells). **J** The expression levels of TNF-α were detected by real-time qPCR. **K** The expression levels of IL-1β were detected by real-time qPCR. **M** The expression levels of TGF-β1 were detected by real-time qPCR. **N** The expression levels of Arg1 were detected by real-time qPCR. Data are expressed as means ± sd (*n* = 6). ^#^*P* < *0.05* versus Scramble. *P < *0.05* versus LPS or IL-4 group.

**Fig. 6:**
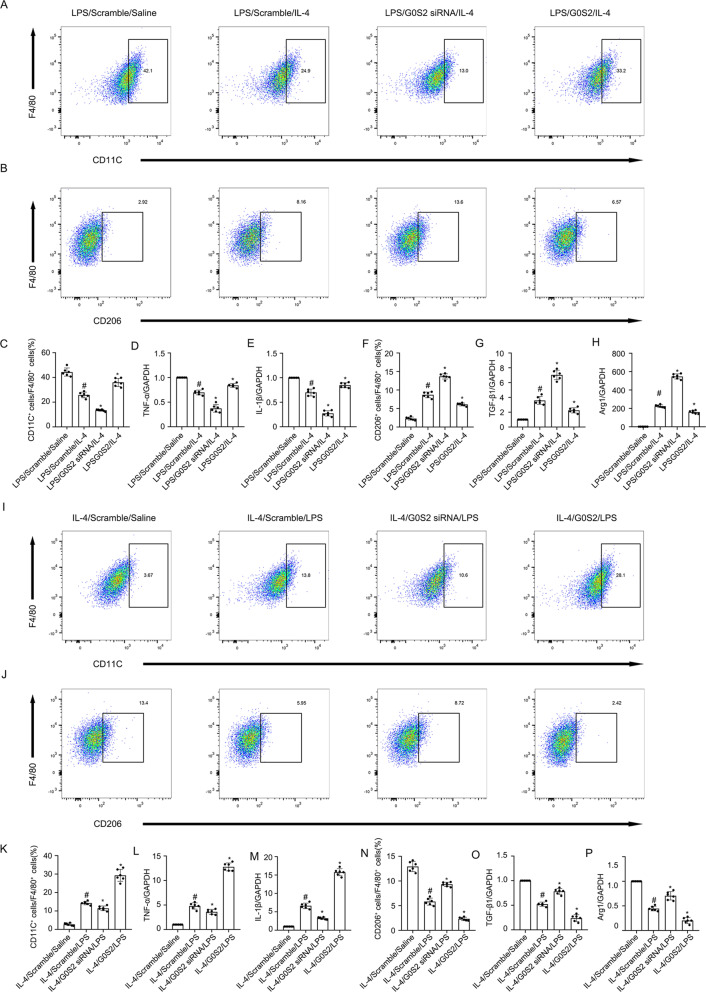
G0S2 promoted transition of M2 to M1. RAW264.7 macrophage cells were transfected with G0S2 siRNA or G0S2 plasmid plus with LPS 1 μg/ml or IL-4 20 ng/ml treatment for 24 h. **A**, **C**, **I**, and **K** Representative FCM analysis the ration of M1 (CD11C^+^ cells vs. F4/80 cells). **B**, **F**, **J**, and **N** Representative FCM analysis the ration of M2 (CD206^+^ cells vs. F4/80 cells). **D** and **L** The expression levels of TNF-α were detected by real-time qPCR. **E** and **M** The expression levels of IL-1β were detected by real-time qPCR. **G** and **O** The expression levels of TGF-β1 were detected by real-time qPCR. **H** and **P** The expression levels of Arg1 were detected by real-time qPCR. ^#^*P* < *0.05* versus Scramble plus LPS or IL-4 group. Data are expressed as means ± sd (*n* = 6). *P < *0.05* versus LPS and IL-4 group.

A recent study demonstrated that G0S2 mediated the infiltration of macrophages into injured kidneys [15]. However, the role of G0S2 in the differentiation of macrophages remains unclear. We demonstrated that G0S2 expression in RAW264.7 macrophages was initiated within 12 h of treatment with LPS or IL-4, peaked at 24 h, and then significantly declined by 48 h (Fig. 5A–D). To verify the effects of the G0S2 plasmid or siRNA, they were transfected into RAW264.7 macrophages. The results indicated that G0S2 expression was enhanced with the application of the G0S2 plasmid, and expression of G0S2 was inhibited with the application of G0S2 siRNA (Fig. 5E, F). RAW264.7 macrophages were transfected with the G0S2 siRNA or plasmid and then stimulated with LPS or IL-4. Subsequent FCM analysis and RT-qPCR results indicated that the G0S2 siRNA prevented the LPS- or IL-4-induced transition of M0 macrophages into M1 or M2 macrophages. On the other hand, G0S2 overexpression had the opposite effect from G0S2 siRNA (Fig. 5G–N). Then, M1 or M2 macrophages were transfected with the G0S2 siRNA or plasmid plus exposed to IL-4 or LPS. Subsequent FCM analysis and RT-qPCR results verified that the G0S2 siRNA promoted the differentiation of M1 macrophages into M2 macrophages. However, G0S2 overexpression had the opposite effect from G0S2 siRNA (Fig. 6A–H). The FCM analysis and RT-qPCR results also demonstrated that the differentiation of M2 macrophages into M1 macrophages was blocked by G0S2 siRNA. In contrast, the effect was enhanced with overexpression of MBD2 (Fig. 6I–P). Collectively, these data suggested that the role of G0S2 in macrophage differentiation was similar to that of MBD2.

### MBD2-LysM^Cre^ mice were constructed

To clarify the role of MBD2 in macrophages, we established a mouse model of MBD2-LysM^Cre^. MBD2^flox/flox^LysM^WT/WT^ males were crossed with MBD2^+/+^LysM^WT/Cre^ females. After two generations, MBD2-LysM^WT^ (MBD2^flox/flox^LysM^WT/WT^, MBD2^flox/+^LysM^WT/WT^), and MBD2-LysM^Cre^ (MBD2^flox/flox^LysM^WT/Cre^) mice were produced (Supplementary Fig. 1A). RT-qPCR results demonstrated that the genotype of the MBD2-LysM^Cre^ mice was characterized by a 213-bp DNA fragment floxed allele, a 350-bp DNA fragment of the WT allele, and a 700-bp DNA fragment of the Cre gene (Supplementary Fig. 1B, lanes 3 and 6). The genotype of the WT (MBD2-LysM^WT^) mice lacked the Cre gene (Supplementary Fig. 1B, lanes 1, 2, and 5). Moreover, FCM analysis demonstrated that the MBD2-LysM^Cre^ mice exhibited substantially reduced UUO-induced infiltration of M1 and M2 macrophages and upregulation of TNF-α and IL-1β (an M1 marker) as well as TGF-β1 and Arg1 (an M2 marker) (Supplementary Fig. 1C–J).

### G0S2 mediated the MBD2-induced macrophage transition

Although we demonstrated that both MBD2 and G0S2 were involved in the macrophage transition (Figs. 2 and 3, 5 and 6), it was not clear whether G0S2 mediated the macrophage transition that was promoted by MBD2. The primary murine bone marrow-derived monocytes from the MBD2-LysM^Cre^ mice and their wild-type MBD2-LysM^WT^ littermates were transformed in vitro into M0 macrophages, then transfected with the G0S2 plasmid plus exposure to LPS or IL-4. The FCM analysis and RT-qPCR results demonstrated that the MBD2-KO prevented LPS or IL-4 from promoting the differentiation of M0 macrophages to M1 or M2 macrophages. On the other hand, this effect was reversed by overexpression of G0S2 (Supplementary Fig. 2A–H). Furthermore, the FCM analysis and RT-qPCR results demonstrated that the MBD2-KO promoted the transition of M1 macrophages to M2 macrophages. However, this effect was almost completely reversed by overexpression of G0S2 (Supplementary Fig. 2I–P). Thus, these data demonstrated that G0S2 mediated the macrophage transition induced by MBD2.

### MBD2 mediated the M1 macrophage-induced renal fibrosis via upregulation of G0S2 in M1 macrophages co-cultured with or without murine embryonic NIH 3T3 fibroblasts

RAW264.7 macrophages were transfected with the MBD2 plasmid, or G0S2 siRNA, or MBD2 siRNA, or G0S2 plasmid plus LPS treatment. After 24 h of culture, the supernatant was collected and the cells were subsequently stimulated with murine embryonic NIH 3T3 fibroblasts for another 24 h (Supplementary Fig. 3A and J). The results indicated that the G0S2 siRNA attenuated the M1 macrophages with the MBD2-induced expression of FN, Col I and IV, and α-SMA in murine embryonic NIH 3T3 fibroblasts (Supplementary Fig. 3B, C). The M1 macrophages transfected with MBD2 siRNA exhibited reduced expression of FN, Col I and IV, and α-SMA in murine embryonic NIH 3T3 fibroblasts. In contrast, this effect was reversed by overexpression of G0S2 (Supplementary Fig. 3D, E). These data suggested that MBD2 mediated the fibrosis caused by the M1 macrophages through the regulation of G0S2.

### MBD2-LysM^Cre^ mice ameliorated the UUO-induced renal fibrosis

Littermates of the MBD2-LysM^WT^ and MBD2-LysM^Cre^ mice were subjected to UUO for seven days. The HE and Masson’s trichome staining indicated that the MBD2-LysM^Cre^ mice exhibited reduced UUO-induced tubular dilation and atrophy and ECM accumulation (Supplementary Fig. 4A, B, and G). The immunohistochemical staining supported the findings associated with Masson’s trichome staining (Supplementary Fig. 4C–F and H). The Western blot results verified that the MBD2-LysM^Cre^ mice exhibited markedly attenuated UUO-induced expression of FN, Col I and IV, and α-SMA through the downregulation of G0S2 (Supplementary Fig. 4I, J). These data supported that macrophage-expressed MBD2 mediated the progression of renal fibrosis during UUO injury.

### I/R-induced renal fibrosis was attenuated by the MBD2-LysM^Cre^ mice

To further confirm the role of macrophages MBD2 in renal fibrosis, ischemic injury model was established in subsequent study. Specifically, littermates of the MBD2-LysM^WT^ and MBD2-LysM^Cre^ mice were subjected to I/R for 21 days. The HE and Masson’s trichome staining indicated that I/R-induced renal function declined, tubular damage, and interstitial fibrosis was markedly attenuated by the MBD2-LysM^Cre^ mice (Supplemental Fig. 5A, B), this was further verified by the immunohistochemical staining of FN, Col I and IV, and α-SMA (Supplemental Fig. 5C–F and H). The immunoblot analysis further verified that the MBD2-LysM^Cre^ mice exhibited markedly attenuated I/R-induced expression of them via the downregulation of G0S2 (Supplemental Fig. 5I, J). These data supported that macrophage MBD2 not only mediated UUO-induced renal fibrosis but also mediated I/R-induced renal fibrosis.

### MBD2 inhibited the expression of p53 and stat3 in RAW264.7 macrophages

Our previous study reported that MBD2 mediated vancomycin (VAN)-induced renal cell apoptosis via upregulation of p53 [18]. Moreover, another study found that DNA methylation was involved in the expression of stat3 [34]. In current study, the immunoblot analysis indicated that knock of MBD2 not only suppressed the LPS induced the expression of p53, but also inhibited the activation and expression of stat3 (Supplementary Fig. 6A, B). Overall, the data demonstrated that MBD2 promoted the expression of p53 as well as activation and expression of stat3 in RAW264.7 macrophages during LPS treatment.

## Discussion

Although MBD2 has been considered the most promising target for DNA demethylation therapy in tumor disease [17, 35], its role in macrophages differentiation and renal fibrosis remained largely unknown. In the present study, for the first time, we demonstrated that MBD2 directly resulted in the increased expression of G0S2 to induce renal fibrosis and transition of M0 macrophages into M1 or M2 macrophages and M2 macrophages into M1 macrophages via suppression of the methylation levels of their promoters, respectively (Supplementary Fig. 7). The creation of the MBD2-LysM^Cre^ mice following UUO and I/R injury further confirmed these findings. Together, these results provided substantial evidence to support the theory that MBD2 in macrophages is a therapeutic target for renal fibrosis.

Recent advances in understanding the functional role of DNA methylation in renal fibrosis have been reported [36]. However, the role of the MBD family in renal fibrosis is unknown. In the current study, we demonstrated that MBD2 was significantly increased in UUO mice. Further, we found that the expression of F4/80 and number of M1 and M2 macrophages in the kidney also reached a peak after three days and declined after seven days (Fig. 1). These data suggested that UUO induced the expression of MBD2 and numbers of M1 and M2 macrophages in kidney.

Previous studies suggested that MBD2 was involved in regulating T cells in immune-related diseases [37–39]. Thus far, the role of MBD2 in the transition of macrophages remains unclear. A new recent study found that MBD2 drives transition of the M0 macrophages into M2 macrophages [12]. In the current study, we revealed that MBD2 not only promoted the differentiation of M0 macrophages into M2 macrophages but also induced them into M1 macrophages, which is revealed by the following evidence. First, MBD2 overexpression promoted the transition of M0 macrophages into M1 or M2 macrophages caused by exposure to LPS or IL-4, respectively. However, MBD2 knockdown prevented this effect (Fig. 2). Secondly, MBD2-LysM^Cre^ mice markedly reduced the number of M1 and M2 macrophages induced in the kidney by UUO and ischemic injury (Supplemental Fig. 4 and 5). Also, we found that MBD2 induced the transition of M2 macrophages to M1 macrophages and prevented the transition of M1 macrophages to M2 macrophages (Fig. 3), which suggested that MBD2 contributed to the increased number of M1 macrophages that were observed during UUO injury.

To investigate the mechanism of MBD2 in macrophage transition, we focused on G0S2. One study suggested that G0S2 promoted the progression of renal inflammation accompanied by F4/80-positive cell infiltration in a 5/6Nx mouse model [15]. However, the role of G0S2 in macrophage transition remained unclear. In the current study, G0S2 was induced by LPS and IL-4 treatment (Fig. 5A–D). Functionally, G0S2 overexpression promoted LPS- or IL-4-induced transition of M0 macrophages into M1 or M2 macrophages, respectively. In contrast, G0S2 siRNA prevented the transition (Fig. 5G–N). Our results also demonstrated that G0S2 promoted the differentiation of M2 macrophages into M1 macrophages and blocked the transition of M1 macrophages into M2 macrophages (Fig. 6). These data suggested that the role of G0S2 in macrophage transition was similar to that of MBD2. However, it was unclear whether G0S2 mediated the action of MBD2 on the macrophage transition. Our data demonstrated that knockdown of MBD2 reduced the differentiation of M0 macrophages into M1 and M2 macrophages, and M1 macrophages into M2 macrophages, and this effect was completely reversed by the overexpression of G0S2 (Supplementary Fig. 2). Co-culture of activated M1 macrophages and murine embryonic NIH 3T3 fibroblasts demonstrated that MBD2 siRNA attenuated M1-induced ECM accumulation by embryonic NIH 3T3 fibroblasts. Furthermore, this effect was reversed by the overexpression of G0S2. On the other hand, the effect of MBD2 overexpression was attenuated by the application of G0S2 siRNA (Supplementary Fig. 3). Thus, our results demonstrated that MBD2 directly induced the expression of G0S2, which was supported by the following evidence. First, ChIP assays indicated that one of five pairs of primers (F5/R5) was amplified. This suggested that MBD2 interacted with this region of the G0S2 promoter, which, in part, verified the predicted result (Fig. 4A, B). Second, MBD2 upregulated the expression of G0S2 through the promotion of hypomethylation of the promoter region (Fig. 4C, D). Finally, MBD2 positively regulated the expression of G0S2 with exposure to LPS or IL-4 (Fig. 4E–L). In addition, we also found that knockdown of MBD2 inhibited LPS induced the expression of p53 as well as activation and expression of stat3 in RAW264.7 macrophages (Supplementary Fig. 6). These data collectively suggested that MBD2 promoted the transition of M0 macrophages to M1 or M2 macrophages and M2 macrophages to M1 macrophages, which accelerated the renal fibrosis in the UUO model by upregulating G0S2.

In summary, to our knowledge, this is the first report that MBD2 in macrophages could act as an inducer of renal fibrosis. This observation was strongly supported by evidence that silencing or deleting MBD2 in macrophages attenuated the UUO and I/R-induced renal fibrosis in mice. Furthermore, we described a novel mechanism by which MBD2 induced the expression of G0S2. MBD2 also directly or indirectly promoted the transition of M0 macrophages into M1 or M2 macrophages and M2 macrophages into M1 macrophages. These changes resulted in increased renal fibrosis by inducing the hypomethylation of the promotor. Thus, this study suggested that MBD2 in macrophages might be an attractive therapeutic target for renal fibrosis.

## Supplementary information

agreement of author list changes

supplementary figures

reproducibility checklist

## Data Availability

The datasets used and/or analyzed during the current study are available from the corresponding author by request.
