# Supplementary material for: Methyl-CpG-binding domain protein 2 contributes to renal fibrosis through promoting polarized M1 macrophages
Source: Cell Death Dis. 2022 Feb 8;13(2):125. doi: 10.1038/s41419-022-04577-3 (PMC8826408; doi:10.1038/s41419-022-04577-3)
Supplement: Supplementary file 2 — supplementary figures [file 41419_2022_4577_MOESM2_ESM.docx]

**Methyl-CpG–binding domain protein 2** **contributes to renal fibrosis through promoting polarized M1 macrophages**

Kai Ai^1,2,3^*, Jian Pan^1,2^*, Pan Zhang^5^*, Huiling Li^4^, Zhibiao He^1,2^, Hongliang Zhang^1,2^, Yijian Li^3^, Lei Yi^3^, Ye Kang^3^, Yinhuai Wang^3^, Xudong Xiang^1,2^, Xiangpin Chai^1,2^, Dongshan Zhang ^1,2^


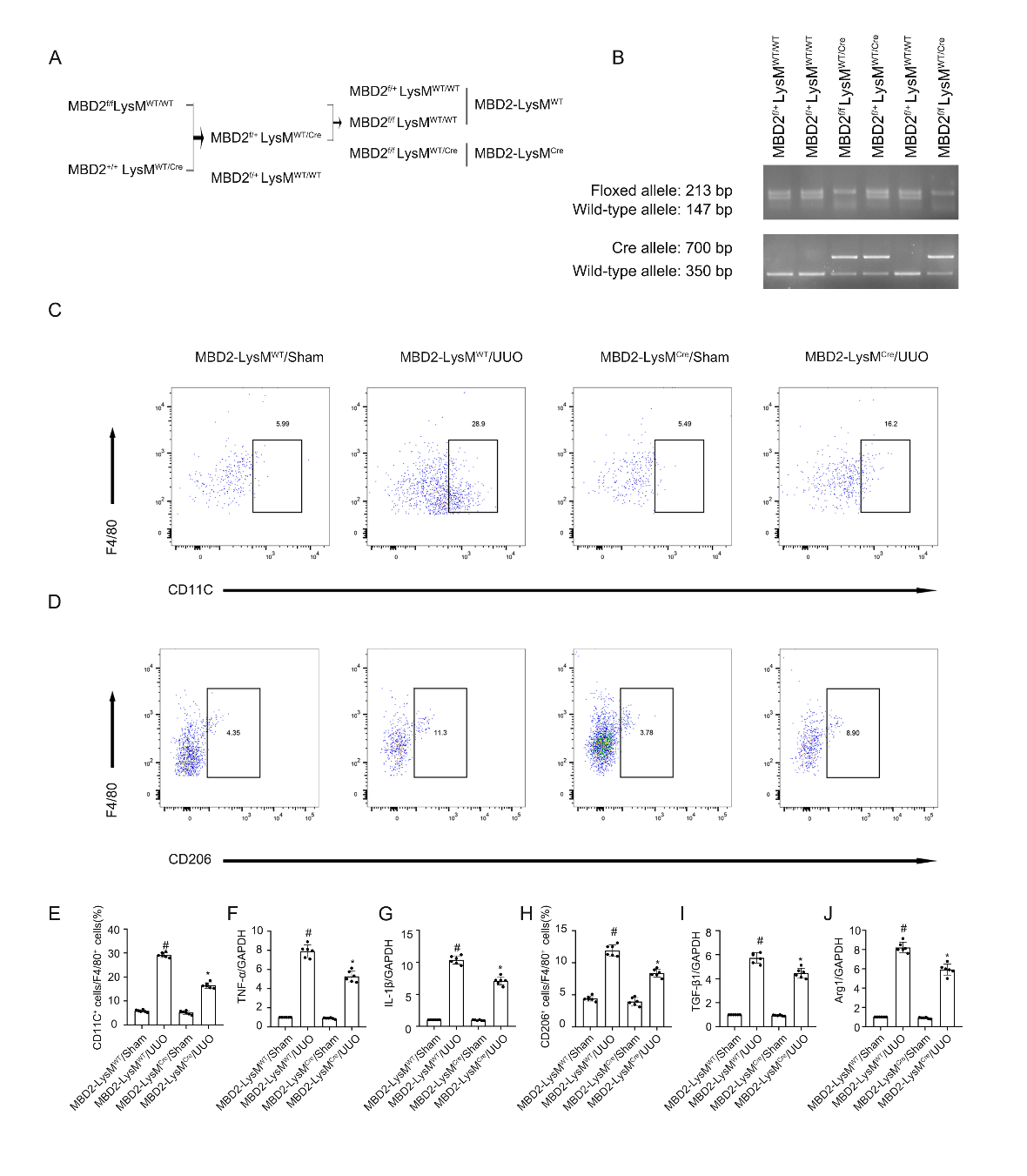


**Supplemental Figure1. Generation and features of the MBD2-LysM^Cre^ mouse model.** (A) Breeding procedure for the creation of MBD2-LysM^Cre^ mice. (B) PCR-based genotyping of wild-type and floxed alleles of MBD2 and LysM-Cre allele. (C&E) Representative FCM analysis the ration of M1 (CD11C+ cells vs. F4/80 cells). (F&G) The expression levels of TNF-α and IL-1β (M1 marker) were detected by real-time qPCR. (D&H) Representative FCM analysis of the ration of M2 (CD206+ cells vs. F4/80 cells). (I&J) The expression levels of TGF-β1 and Arg1 were detected by real-time qPCR. Data are expressed as means ± s.d. (n= 6). # *P < 0.05* versus sham group. **P < 0.05* versus MBD2-LysM^WT^ with UUO group.


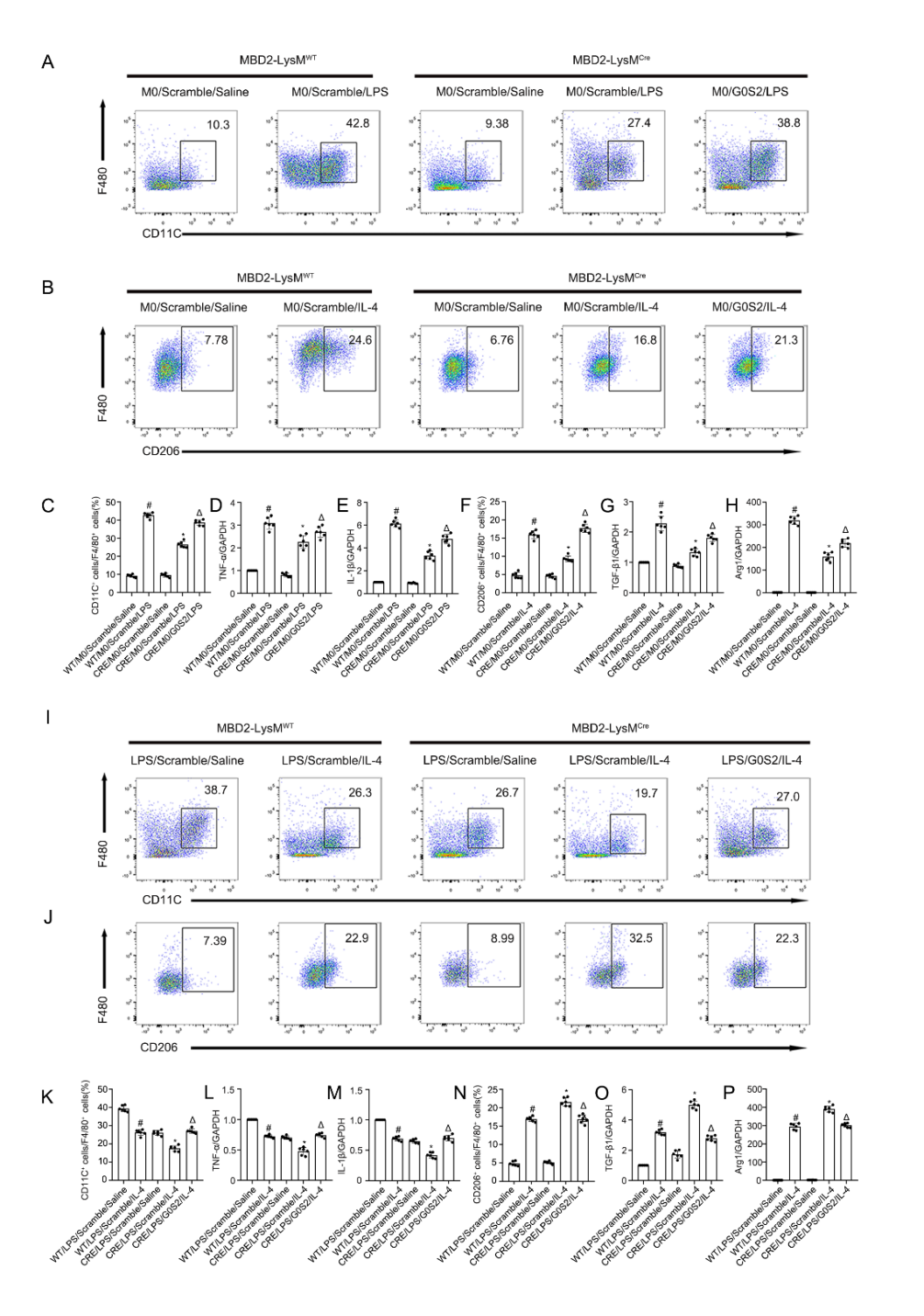


**Supplemental Figure2. The effects of MBD2-LysM^Cre^ on the transition of M0 macrophages to M1 or M2, and M2 to M1 was reversed by overexpression of G0S2.** The monocytes from primary murine bone marrow of MBD2-LysM^Cre^ mice and MBD2-LysM^WT^ littermates were transited in vitro into M0 macrophages, and then transfected with G0S2 plasmid plus with LPS or IL-4 treatment. (A, C, I and K) Representative FCM analysis the ration of M1 (CD11C^+^ cells vs. F4/80 cells). (B, F, J and N) Representative FCM analysis the ration of M2 (CD206^+^ cells vs. F4/80 cells). (D & L) The expression levels of TNF-α were detected by real-time qPCR. (E & M) The expression levels of IL-1β were detected by real-time qPCR. (G & O) The expression levels of TGF-β1 were detected by real-time qPCR. (H & P) The expression levels of Arg1 were detected by real-time qPCR. # *P<0.05* versus MBD2-LysM^WT^ -M0 with Scramble plus with or without LPS. * *P<0.05* versus MBD2-LysM^WT^ -M0 with LPS or IL-4, or LPS/Scramble/IL-4 group, ∆ *P<0.05* versus MBD2-LysM^Cre^ M0/Scramble/LPS or IL-4, or MBD2-LysM^Cre^ with LPS/Scramble/IL-4 group.


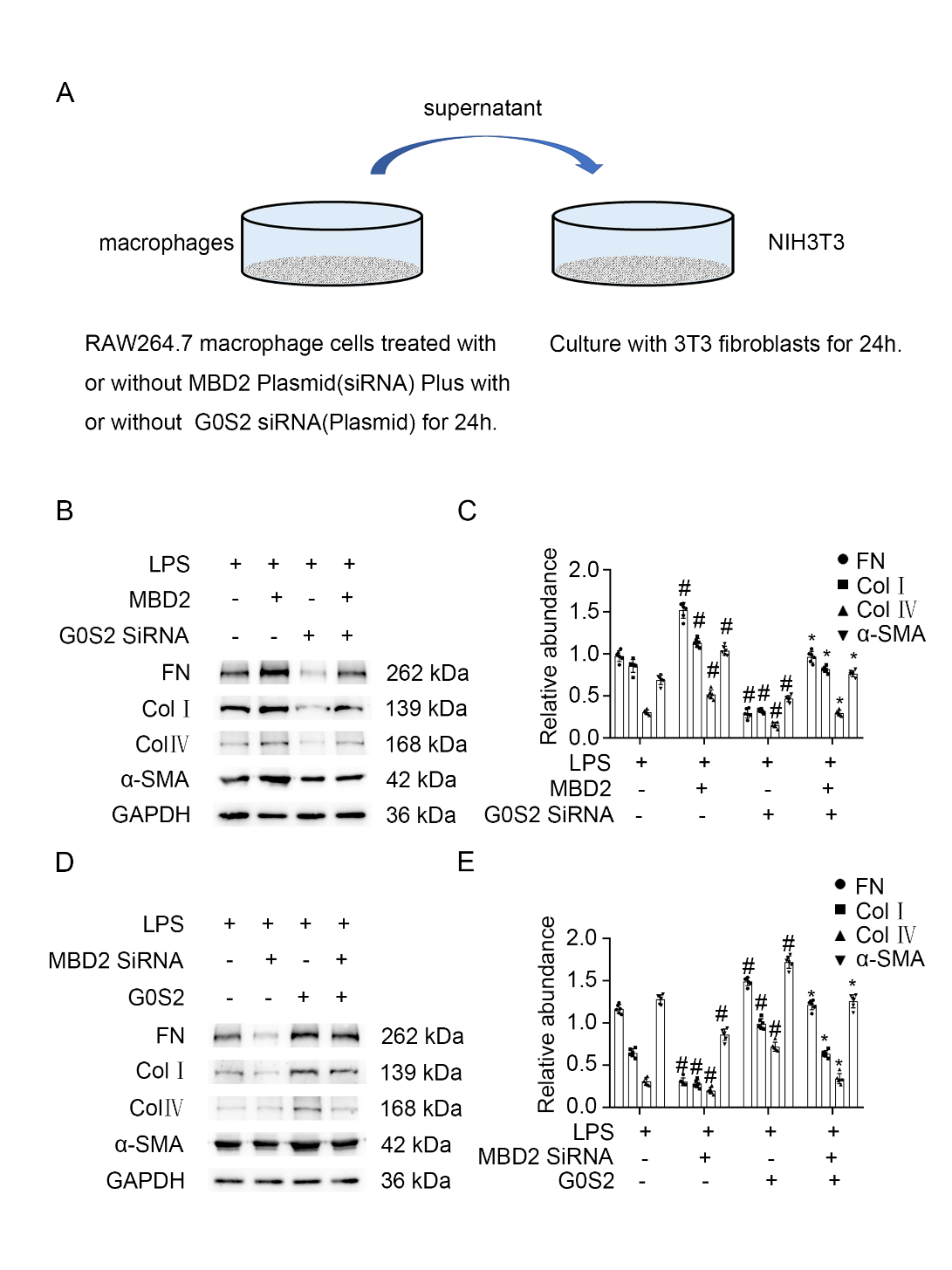


**Supplemental Figure3. G0S2-mediated the promoting fibrosis role of MBD2 during co-culture of murine embryonic NIH 3T3 fibroblasts and RAW264.7 macrophage cells transfected with MBD2 plus with LPS**. RAW264.7 macrophage cells were transfected with MBD2 or G0S2 plasmid or MBD2 or G0S2 siRNA plus with LPS treatment for 24h, the supernatant of them were collected to treat murine embryonic NIH 3T3 fibroblasts for another 24h. (A) The co-culture model diagram of M0 macrophages and murine embryonic NIH 3T3 fibroblasts. (B&D) Immunoblot analysis of FN, and Col I&IV, and α-SMA. (C&E) Analysis of the grayscale image between them. Data are expressed as means ± sd (n=6). # *P<0.05* versus Scramble with LPS group. **P<0.05* versus LPS with MBD2 plasmid or MBD2 siRNA group


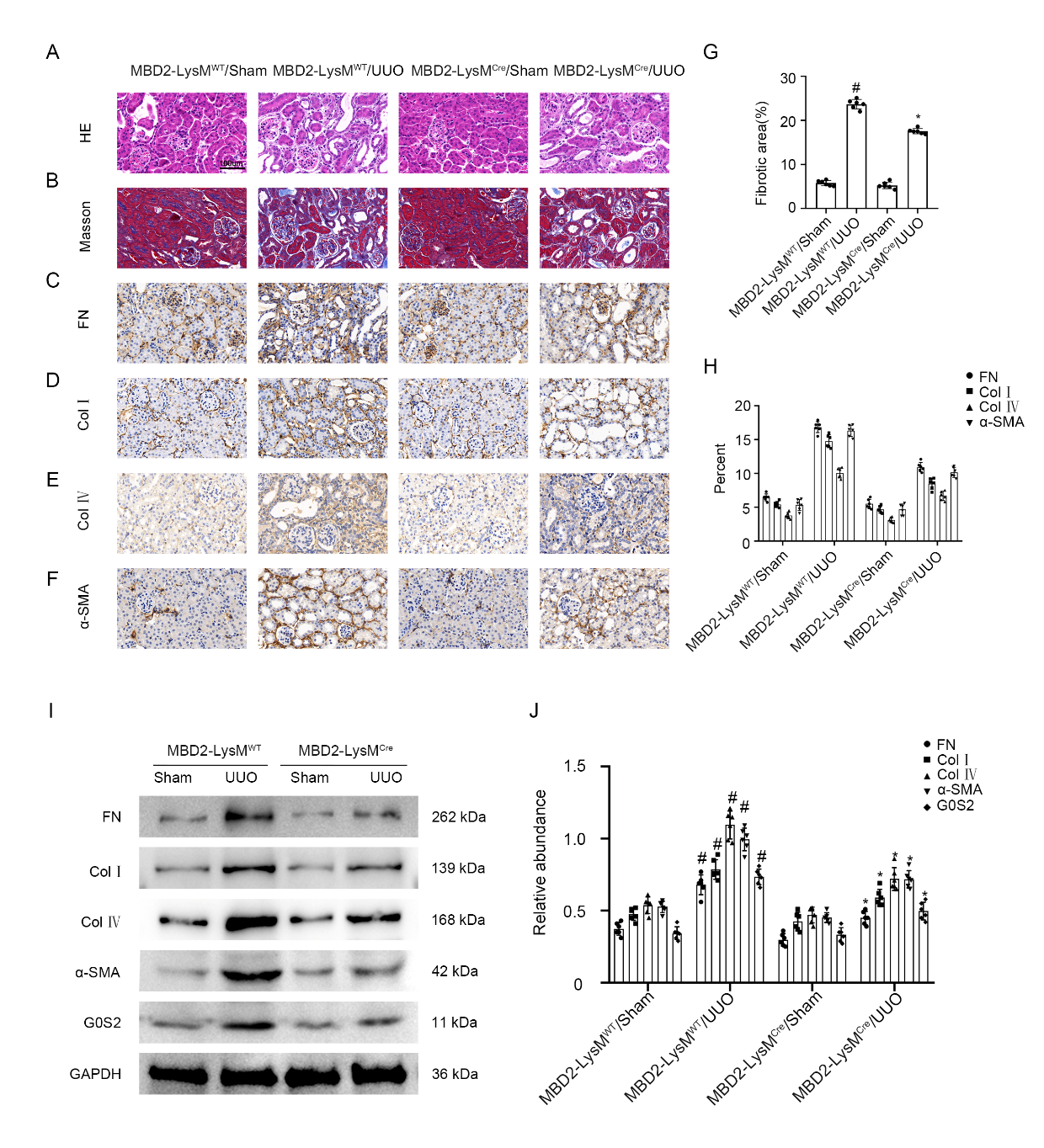


**Supplemental Figure4.** **MBD2-LysM^Cre^ mice attenuated UUO-induced renal fibrosis.** The left ureter of MBD2-LysM^WT^ and MBD2-LysM^Cre^ littermate mice was ligated to establish UUO model for 7 days. (A) Representative hematoxylin and eosin staining. (B) Masson trichrome staining shows interstitial collagen deposition (blue). (C-F) Immunohistochemistry staining of FN, Col I, Col IV, and α-SMA. (G) Quantify tubulointerstitial fibrosis in the kidney cortex. (H) Quantification of immunohistochemistry staining. (I) Representative immunoblots for the expression of FN, Col I, Col IV, α-SMA, G0S2, and GAPDH. (J) Densitometry analysis of proteins signals, and normalized to internal control of GAPDH. Data are expressed as means ± sd (n=6). # *P < 0.05* versus sham group. **P < 0.05* versus MBD2-LysM^WT^ with UUO group. Original magnification, x400.


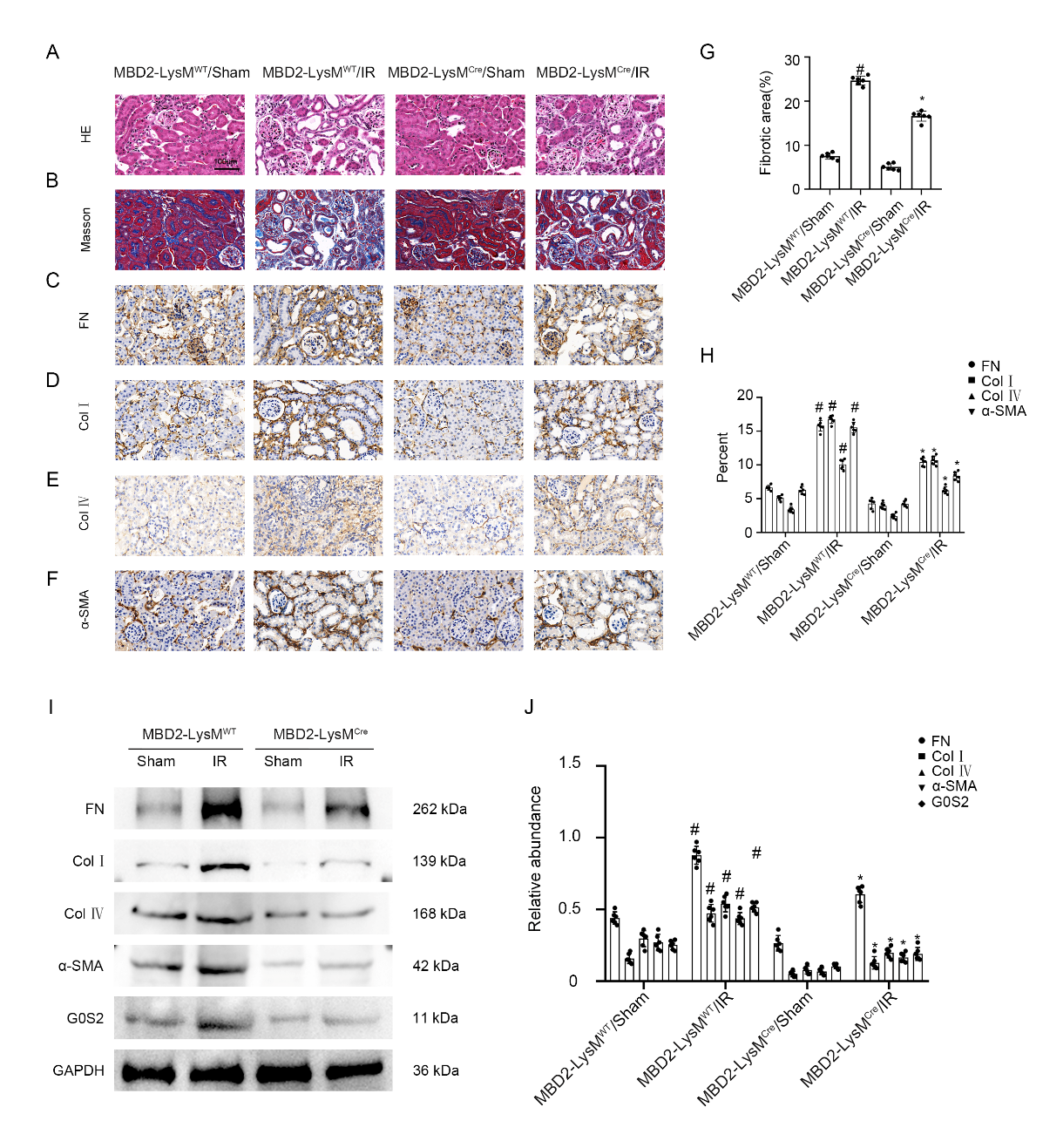


**Supplemental Figure5. MBD2-LysM^Cre^ mice attenuated IR-induced renal fibrosis.** The littermate mice of the MBD2-LysMWT and MBD2-LysMCre were subjected to 28 minutes of bilateral renal ischemia followed by 21 days of reperfusion. (A) Representative hematoxylin and eosin staining. (B) Masson trichrome staining shows interstitial collagen deposition (blue). (C-F) Immunohistochemistry staining of FN, Col I, Col IV, and α-SMA. (G) Quantify tubulointerstitial fibrosis in the kidney cortex. (H) Quantification of immunohistochemistry staining. (I) Representative immunoblots for the expression of FN, Col I, Col IV, α-SMA, G0S2, and GAPDH. (J) Densitometry analysis of proteins signals, and normalized to internal control of GAPDH. Data are expressed as means ± sd (n=6). # *P < 0.05* versus sham group. **P < 0.05* versus MBD2-LysM^WT^ with I/R group. Original magnification, x400.


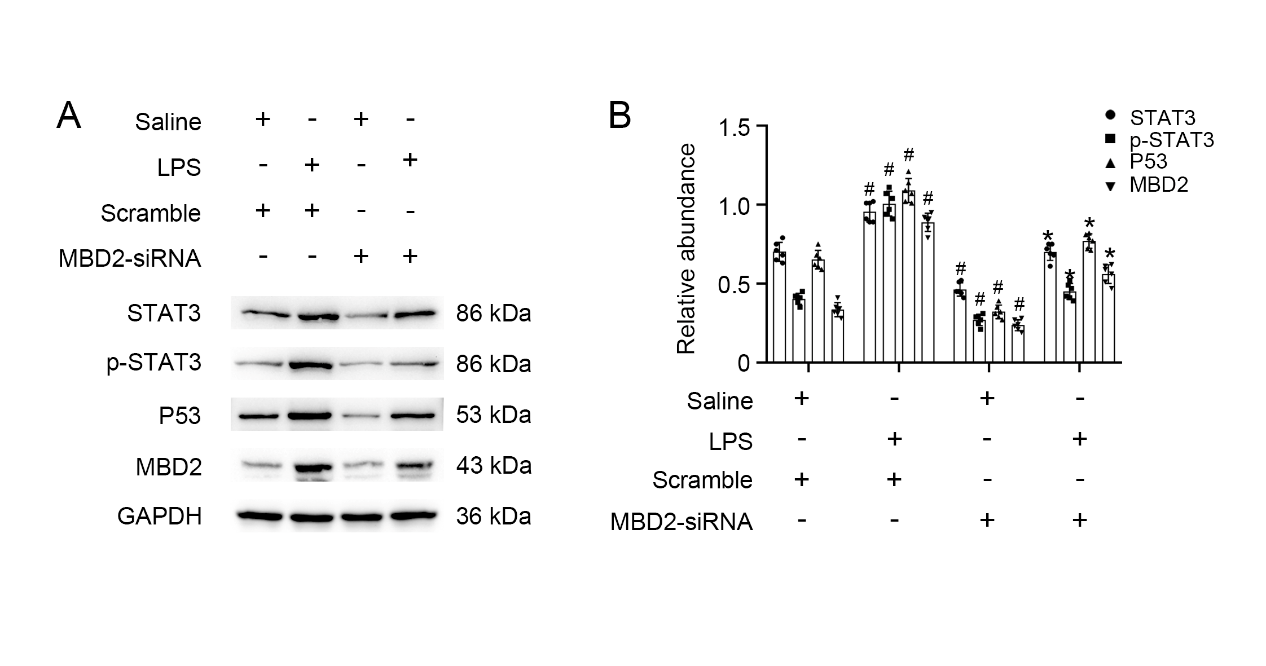


**Supplemental Figure6.** MBD2 mediated LPS-induced the expression of p53 and stat3 in RAW264.7 macrophages. RAW264.7 macrophage cells were transfected with MBD2 siRNA plus with LPS treatment for 24h. (A) Immunoblot analysis of the expression of STAT3, p-STAT3, P53, and MBD2. (B) Densitometry analysis of proteins levels, and normalized to internal control of GAPDH. Data are expressed as means ± sd (n=6). # *P<0.05* versus Saline with Scramble group. **P<0.05* versus LPS with Scramble group.


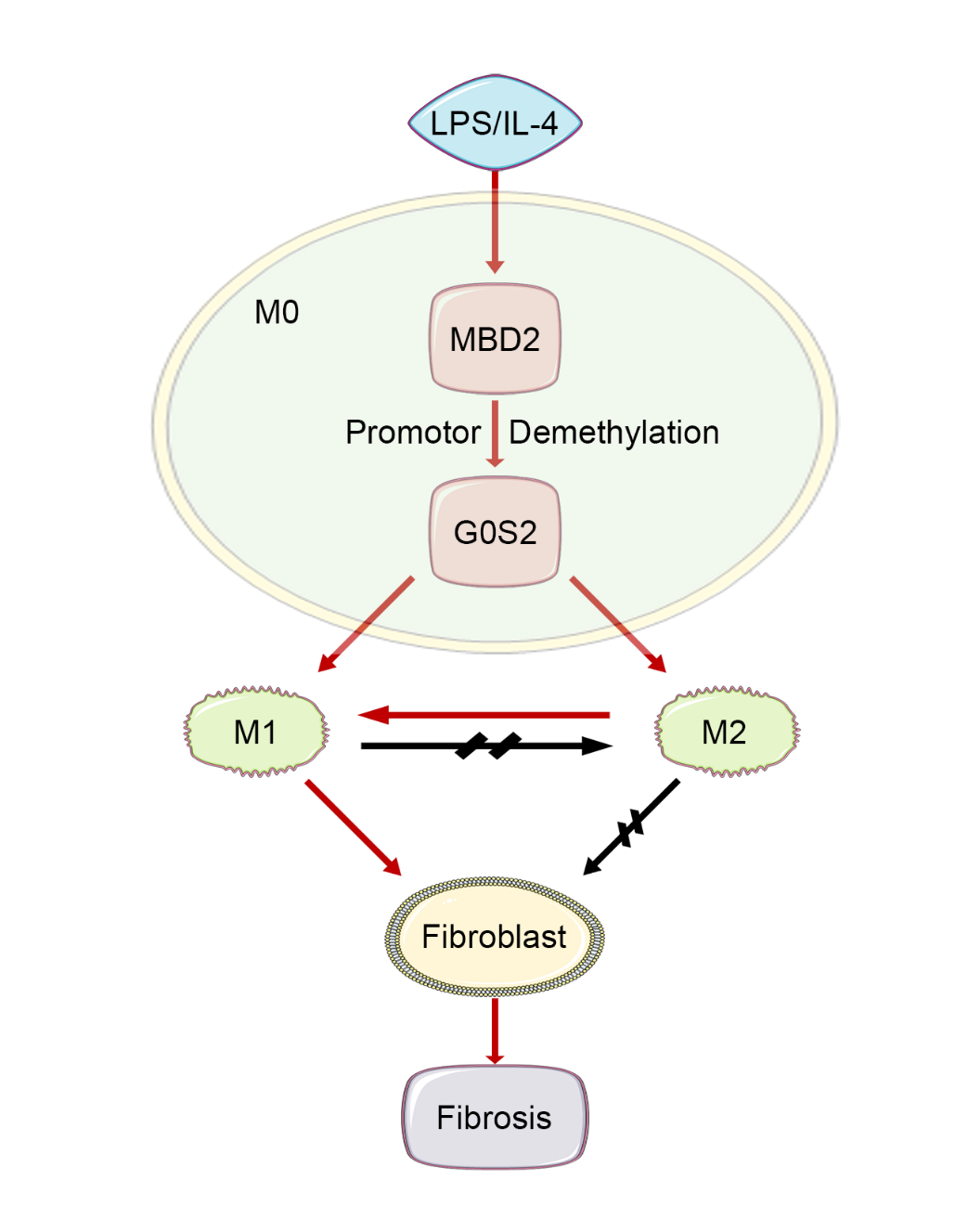


**Supplemental Figure7.** The role and molecular mechanism of macrophages MBD2 in renal fibrosis. After LPS/IL-4 or TGF-β1 treatment, MBD2 upregulates G0S2 expression by hypomethylation of promoter, and then and then directly or indirectly (promote the transition of M0 to M1 or M2 and M2 to M1) increase the renal fibrosis.
