# Supplementary material for: Methyl-CpG-binding domain protein 2 contributes to renal fibrosis through promoting polarized M1 macrophages
Source: Cell Death Dis. 2022 Feb 8;13(2):125. doi: 10.1038/s41419-022-04577-3 (PMC8826408; doi:10.1038/s41419-022-04577-3)
Supplement: Supplementary file 1 — agreement of author list changes [file 41419_2022_4577_MOESM1_ESM.pdf]

Re: Confirm author changes ☆
发件人：**aikai2015** <aikai2015@csu.edu.cn>
时　间：2022年1月11日（星期二）下午11：13
收件人：誓言 <zhkidney@qq.com>

这不是腾讯公司的官方邮件。请勿轻信密保、汇款、中奖信息，勿轻易拨打陌生电话。举报垃圾邮件 网站安全云检测

邮件可翻译为中文 立即翻译

Dear Dongshan Zhang

Yes, I agree with these changes of author list.

Sincerely

Kai Ai

发自我的iPhone

------------------ Original ------------------

From: zhkidney <zhkidney@qq.com>
Date: Tue,Jan 11,2022 3:23 PM
To: aikai2015 <aikai2015@csu.edu.cn>
Subject: Re: Confirm author changes

Dear Kai Ai,

The manuscript titled"Methyl-CpG-binding domain protein 2 contributes to renal fibrosis through promoting polarized M1 macrophages"has been submitted to the journal of Cell Death & Disease. According to their contributions to the revised manuscript, I added the authors Xiaozhou Li and Yijian Li to the author list. Now I'm writing this email to confirm that whether you agree with this change to the author list.

Hope to hear from you as soon as possible, thank you.

Best wishes

Dongshan Zhang

回复：Confirm author changes ☆
发件人：**hbyhbj** <hbyhbj@qq.com>
时　间：2022年1月11日（星期二）下午4：01
收件人：誓言 <zhkidney@qq.com>

纯文本 |

邮件可翻译为中文 立即翻译

Dear Professor Zhang,
I agree the author change. Thanks for your letter.
sincerely,
Jian Pan

原始邮件

发件人："誓言" <zhkidney@qq.com>;
发送时间：2022年1月11日(星期二) 下午3:24
收件人："Annmarie."<hbyhbj@qq.com>;
主题：Confirm author changes

Dear JianPan,

The manuscript titled "Methyl-CpG-binding domain protein 2 contributes to renal fibrosis through promoting polarized M1 macrophages" has been submitted to the journal of Cell Death & Disease. According to their contributions to the revised manuscript, I added the authors Xiaozhou Li and Yijian Li to the author list. Now I'm writing this email to confirm that whether you agree with this change to the author list.

Hope to hear from you as soon as possible, thank you.

Best wishes

Dongshan Zhang

Re:Confirm author changes ☆
发件人：**cmupaulazhang** <cmupaulazhang@163.com>
时　间：2022年1月11日（星期二）下午3：41
收件人：誓言 <zhkidney@qq.com>

这不是腾讯公司的官方邮件。请勿轻信密保、汇款、中奖信息，勿轻易拨打陌生电话。举报垃圾邮件 网站安全云检测

邮件可翻译为中文 立即翻译

Dear prof. Dongshan Zhang,
I agree with this change to the author list.

Best wishes

Pan Zhang

At 2022-01-11 15:30:20, "誓言" <zhkidney@qq.com> wrote:

Dear Pan Zhang,

The manuscript titled "Methyl-CpG-binding domain protein 2 contributes to renal fibrosis through promoting polarized M1 macrophages" has been submitted to the journal of Cell Death & Disease. According to their contributions to the revised manuscript, I added the authors Xiaozhou Li and Yijian Li to the author list. Now I'm writing this email to confirm that whether you agree with this change to the author list.

Hope to hear from you as soon as possible, thank you.

Best wishes

Dongshan Zhang

Re: Confirm author changes ☆
发件人：**lihuiling** <lihuiling@csu.edu.cn>
时　间：2022年1月11日（星期二）下午3：37
收件人：誓言 <zhkidney@qq.com>

纯文本 |

邮件可翻译为中文 立即翻译

Agree with author changes!

2022-01-11 15:35:59 "誓言" <zhkidney@qq.com> 写道:

Dear Huiling Li,

The manuscript titled "Methyl-CpG-binding domain protein 2 contributes to renal fibrosis through promoting polarized M1 macrophages" has been submitted to the journal of Cell Death & Disease. According to their contributions to the revised manuscript, I added the authors Xiaozhou Li and Yijian Li to the author list. Now I'm writing this email to confirm that whether you agree with this change to the author list.

Hope to hear from you as soon as possible, thank you.

Best wishes

Dongshan Zhang

Re:Confirm author changes

发件人：[illegible] <zbhe267@163.com>
时　间：2022年1月12日（星期三）上午11：50
收件人：警言 <zhkidney@qq.com>

纯文本

邮件可翻译为中文　立即翻译

Dear Dongshan Zhang,

I have received your email ,and agree with the change to the author list.

Best wishes

Zhibiao He

At 2022-01-12 11:36:30, "警言" <zhkidney@qq.com> wrote:

> Dear Zhibiao He,
>
> The manuscript titled "Methyl-CpG-binding domain protein 2 contributes to renal fibrosis through promoting polarized M1 macrophages" has been submitted to the journal of Cell Death & Disease. According to their contributions to the revised manuscript, I added the authors Xiaozhou Li and Yijian Li to the author list. Now I'm writing this email to confirm that whether you agree with this change to the author list.
>
> Hope to hear from you as soon as possible, thank you.
>
> Best wishes
>
> Dongshan Zhang

Re: Confirm author changes

发件人：Hongliang Zhang <zhlzzj@163.com>
时　间：2022年1月11日（星期二）下午4：42
收件人：警言 <zhkidney@qq.com>

这不是腾讯公司的官方邮件。请勿轻信密保、汇款、中奖信息，勿轻易拨打陌生电话。举报垃圾邮件　网站安全云检测

邮件可翻译为中文　立即翻译

好的，没问题

发自我的iPhone

在 2022年1月11日，15:34，警言 <zhkidney@qq.com> 写道:

> Dear Hongliang Zhang,
>
> The manuscript titled "Methyl-CpG-binding domain protein 2 contributes to renal fibrosis through promoting polarized M1 macrophages" has been submitted to the journal of Cell Death & Disease. According to their contributions to the revised manuscript, I added the authors Xiaozhou Li and Yijian Li to the author list. Now I'm writing this email to confirm that whether you agree with this change to the author list.
>
> Hope to hear from you as soon as possible, thank you.
>
> Best wishes
>
> Dongshan Zhang

回复：Confirm author changes

发件人：李晓洲 <467208699@qq.com>
时　间：2022年1月12日（星期三）上午9：37
收件人：警言 <zhkidney@qq.com>

纯文本

邮件可翻译为中文　立即翻译

I agree with this change to the author list.

------------------ 原始邮件 ------------------

发件人: "警言" <zhkidney@qq.com>;
发送时间: 2022年1月11日(星期二) 下午3:35
收件人: "李晓洲"<467208699@qq.com>;
主题: Confirm author changes

Dear Xiaozhou Li,

The manuscript titled "Methyl-CpG-binding domain protein 2 contributes to renal fibrosis through promoting polarized M1 macrophages" has been submitted to the journal of Cell Death & Disease. According to their contributions to the revised manuscript, I added the authors Xiaozhou Li and Yijian Li to the author list. Now I'm writing this email to confirm that whether you agree with this change to the author list.

Hope to hear from you as soon as possible, thank you.

Best wishes

Dongshan Zhang

Re:Confirm author changes

发件人：lyj <laolxy@163.com>
时　间：2022年1月11日（星期二）下午3：53
收件人：警言 <zhkidney@qq.com>

纯文本

邮件可翻译为中文　立即翻译

Dear Dongshan Zhang:
Yes, I agree with this change to the author list.
Yijian Li

At 2022-01-11 15:35:36, "警言" <zhkidney@qq.com> wrote:

> Dear Yijian Li,
>
> The manuscript titled "Methyl-CpG-binding domain protein 2 contributes to renal fibrosis through promoting polarized M1 macrophages" has been submitted to the journal of Cell Death & Disease. According to their contributions to the revised manuscript, I added the authors Xiaozhou Li and Yijian Li to the author list. Now I'm writing this email to confirm that whether you agree with this change to the author list.
>
> Hope to hear from you as soon as possible, thank you.
>
> Best wishes
>
> Dongshan Zhang

Re: Confirm author changes ☆

发件人: **736596534** <736596534@qq.com>
时　间: 2022年1月12日 (星期三) 上午10：23
收件人: 誓言 <zhkidney@qq.com>

纯文本 |

邮件可翻译为中文 立即翻译

Yes, I agree with this change to the author list.

---Original---

From: "誓言"<zhkidney@qq.com>
Date: Tue, Jan 11, 2022 15:37 PM
To: "736596534"<736596534@qq.com>;
Subject: Confirm author changes

Dear Lei Yi,

The manuscript titled"Methyl-CpG-binding domain protein 2 contributes to renal fibrosis through promoting polarized M1 macrophages"has been submitted to the journal of Cell Death & Disease. According to their contributions to the revised manuscript, I added the authors Xiaozhou Li and Yijian Li to the author list. Now I'm writing this email to confirm that whether you agree with this change to the author list.

Hope to hear from you as soon as possible, thank you.

Best wishes

回复: Confirm author changes ☆

发件人: **wangyinhuai** <wangyinhuai@163.com>
时　间: 2022年1月12日 (星期三) 下午4：07
收件人: 誓言 <zhkidney@qq.com>

纯文本 |

邮件可翻译为中文 立即翻译

I agree with the changes of authors.
Dr. Yinhuai Wang

发自我的华为手机

-------- 原始邮件 --------
发件人: 誓言 <zhkidney@qq.com>
日期: 2022年1月11日周二 下午3:40
收件人: wangyinhuai <wangyinhuai@163.com>
主 题: Confirm author changes

Dear Yinhuai Wang,

The manuscript titled"Methyl-CpG-binding domain protein 2 contributes to renal fibrosis through promoting polarized M1 macrophages"has been submitted to the journal of Cell Death & Disease. According to their contributions to the revised manuscript, I added the authors Xiaozhou Li and Yijian Li to the author list. Now I'm writing this email to confirm that whether you agree with this change to the author list.

Hope to hear from you as soon as possible, thank you.

Best wishes

Dongshan Zhang

Re: Confirm author changes ☆

发件人: **ky178211080** <ky178211080@csu.edu.cn>
时　间: 2022年1月11日 (星期二) 下午3：55
收件人: 誓言 <zhkidney@qq.com>

这不是腾讯公司的官方邮件⑦。 请勿轻信密保、汇款、中奖信息，勿轻易拨打陌生电话。 举报垃圾邮件　网站安全云检测

邮件可翻译为中文 立即翻译

Dear Dongshan Zhang:
Yes, I agree with this change to the author list.
Sincerely
Ye Kang

2022-01-11 15:39:10 "誓言" <zhkidney@qq.com> 写道:

Dear Ye Kang,

The manuscript titled "Methyl-CpG-binding domain protein 2 contributes to renal fibrosis through promoting polarized M1 macrophages" has been submitted to the journal of Cell Death & Disease. According to their contributions to the revised manuscript, I added the authors Xiaozhou Li and Yijian Li to the author list. Now I'm writing this email to confirm that whether you agree with this change to the author list.

Hope to hear from you as soon as possible, thank you.

Best wishes

Dongshan Zhang

回复: Confirm author changes ☆

发件人: **xudongx_99** <xudongx_99@hotmail.com>
时 间: 2022年1月11日（星期二）下午9：16
收件人: 誓言 <zhkidney@qq.com>

邮件可翻译为中文 立即翻译

I agree with this change to the author list.

发自我的华为手机

-------- 原始邮件 --------
发件人: 誓言 <zhkidney@qq.com>
日期: 2022年1月11日周二 下午3:40
收件人: xudongx_99 <xudongx_99@hotmail.com>
主 题: Confirm author changes

Dear Xudong Xiang,

The manuscript titled "Methyl-CpG-binding domain protein 2 contributes to renal fibrosis through promoting polarized M1 macrophages" has been submitted to the journal of Cell Death & Disease. According to their contributions to the revised manuscript, I added the authors Xiaozhou Li and Yijian Li to the author list. Now I'm writing this email to confirm that whether you agree with this change to the author list.

Hope to hear from you as soon as possible, thank you.

Best wishes

Dongshen Zhang

Re: Confirm author changes ☆

发件人: **chaixiangping** <chaixiangping@csu.edu.cn>
时 间: 2022年1月12日（星期三）上午9：50
收件人: 誓言 <zhkidney@qq.com>

纯文本

邮件可翻译为中文 立即翻译

Dear Dongshan,

I agree with this change to the author list.

Best,

Xiangping Chai

-----原始邮件-----
发件人:"誓言" <zhkidney@qq.com>
发送时间:2022-01-11 15:41:52 (星期二)
收件人: chaixiangping <chaixiangping@csu.edu.cn>
抄送:
主题: Confirm author changes

Dear Xiangping Chai,

The manuscript titled "Methyl-CpG-binding domain protein 2 contributes to renal fibrosis through promoting polarized M1 macrophages" has been submitted to the journal of Cell Death & Disease. According to their contributions to the revised manuscript, I added the authors Xiaozhou Li and Yijian Li to the author list. Now I'm writing this email to confirm that whether you agree with this change to the author list.

Hope to hear from you as soon as possible, thank you.

Best wishes

Dongshan Zhang
